# Species co-occurrence and management intensity modulate habitat preferences of forest birds

**DOI:** 10.1186/s12915-021-01136-8

**Published:** 2021-09-23

**Authors:** Marco Basile, Thomas Asbeck, João M. Cordeiro Pereira, Grzegorz Mikusiński, Ilse Storch

**Affiliations:** 1 Chair of Wildlife Ecology and Management, University of Freiburg, Tennenbacher Str. 4, 79106 Freiburg, Germany; 2 Swiss Federal Research Institute WSL, Zürcherstrasse 111, 8903 Birmensdorf, Switzerland; 3 Swiss Ornithological Institute, Seerose 1, 6204 Sempach, Switzerland; 4 Chair of Silviculture, University of Freiburg, Tennenbacher Str. 4, 79106 Freiburg, Germany; 5 Grimsö Wildlife Research Station, Department of Ecology, Swedish University of Agricultural Sciences SLU, SE-730 91 Riddarhyttan, Sweden; 6 School for Forest Management, Swedish University of Agricultural Sciences SLU, SE-739 21 Skinnskatteberg, Sweden

**Keywords:** Canopy forager, Cavity nester, Landscape, Multi-species abundance models, Forest management intensity, Temperate forests

## Abstract

**Background:**

Species co-occurrences can have profound effects on the habitat use of species, and therefore habitat structure alone cannot fully explain observed abundances. To account for this aspect of community organization, we developed multi-species abundance models, incorporating the local effect of co-occurring and potentially associated species, alongside with environmental predictors, linked mainly to forest management intensity. We coupled it with a landscape-scale analysis to further examine the role of management intensity in modifying the habitat preferences in connection with the landscape context. Using empirical data from the Black Forest in southern Germany, we focused on the forest bird assemblage and in particular on the cavity-nesting and canopy-foraging guilds. We included in the analysis species that co-occur and for which evidence suggests association is likely.

**Results:**

Our findings show that the local effect of species associations can mitigate the effects of management intensity on forest birds. We also found that bird species express wider habitat preferences in forests under higher management intensity, depending on the landscape context.

**Conclusions:**

We suspect that species associations may facilitate the utilization of a broader range of environmental conditions under intensive forest management, which benefits some species over others. Networks of associations may be a relevant factor in the effectiveness of conservation-oriented forest management.

**Supplementary Information:**

The online version contains supplementary material available at 10.1186/s12915-021-01136-8.

## Background

Species assemblages form as a product of environmental filtering (i.e., local environmental conditions acting as filters for local species sorting) and species interactions [1]. In forest ecosystems, environmental filtering depends, among other factors, on the main tree species, which can be subject to spatial and temporal variation [2]. Trees may alter the biotic conditions and modulate the access to resources for other species. For instance, trees can modify the amount of light that reaches lower forest layers and profoundly impact the plant species composing the herb layer [3] or change micro-morphological and chemical soil properties and greatly affect soil organisms [4]. Similarly, the occurrence and abundance of forest-inhabiting taxa such as bats, birds or insects may depend on tree structures such as rot holes and cavities providing resources to them [5–7].

In contrast, species interactions are based on intra- and interspecific competition for resources [8], along with other trophic interactions such as predation [9] or facilitative interactions [10]. Species interactions are often simplistically assumed based on patterns of species co-occurrences, in relation to a baseline occurrence rate dependent on the environmental conditions [11]. Despite criticism to this approach [12], the inclusion of an interaction component can indeed improve the outcome of ecological niche modeling [13]. In this context, however, interactions are reduced to co-occurrences, which may, in turn, be non-random and linked to unknown processes which cause species associations. Yet the effect of interacting species can modify ecological niches within populations, highlighting inter-individual differences [14], or differences within meta-populations, e.g., following the introduction of allochthonous species [15].

The concomitance of biotic and abiotic factors and processes shapes the ecological niche of species, described as the Hutchinsonian niche [16, 17]. This is further modulated by anthropogenic influences that often elicit different local responses within species’ distribution ranges, frequently leading to niche contraction and population decline when anthropogenic disturbance is dominant [18]. In the case of forests, forestry can be a fundamental determinant of the habitat use for forest species, hence altering their ecological niche [19]. Birds are affected by forestry operations at the level of individuals, populations, and communities, through habitat simplification, which deprives birds of important resources such as suitable nest sites or food supply [20–23]. However, landscape characteristics can contribute to alter the realized niche, despite the habitat conditions. For instance, the degree of forest fragmentation can affect the ability of species to spill over into suboptimal habitat types, a density-dependent process also influenced by regional abundances [24]. This often results in local niche contractions [24], as the overall breadth of the realized niches is related to the ability of species to cope with the landscape context and colonize different habitat patches [25, 26].

Some of the bird species most affected by forestry belong to the cavity-nesting guild [27], which is composed by primary cavity nesters, i.e., those bird species that excavate their own cavities in trees, and secondary cavity nesters, i.e., those that use cavities generated by natural processes or excavated by primary cavity nesters [28]. The substrate (trees and snags with specific characteristics) where the cavity is located may trigger competitive interactions among species [29, 30]. Experimental evidence indicates that the supply of cavities can limit the number of cavity nesters [28, 31]. Forest management for timber production is considered one of the main causes of shortages in cavity supply [32], usually reflected by a relative decline of the cavity-nesting guild within the bird assemblage [27]. The main reason is that such management leads to a shortage of trees or snags with characteristics enabling formation of natural cavities or allowing their excavation by primary cavity nesters [33]. This is in contrast to primeval forests with plentiful decay-formed cavities, where the abundance of nest sites is not a limiting factor for cavity nesters and woodpeckers are not always the key cavity providers [34]. Hence, in managed forests species-specific responses to forestry operations might show a high degree of variation depending on the relative abundance of large, suitable trees, and snags offering the potential to provide cavities [35, 36], the relative abundance of woodpeckers [37], the forest management type [38], and the tree species composition [39]. Moreover, the predation risk [40, 41] and the presence of invasive species [42] are additional factors shaping the cavity-nesting bird guild. Considering all those factors, we could expect that the abundance of cavity nesters is regulated by the relative abundance of each species included in this guild, via competition for resources, interference, facilitation, or to a lesser extent, predation.

Another bird guild highly influenced by forestry is the canopy-foraging guild [43–45], which includes those species that feed on substrates in the tree canopy [46]. In this case, competition sparks from the optimal foraging substrate (e.g., large branches vs. small branches with needles in conifer canopy) and the different efficiency of each species’ foraging technique [47–49]. In Europe, it comprises mainly foliage gleaners and seed eaters. In the case of canopy foragers, the main interaction we could expect is resource competition, where the presence of a given species reduces the foraging efficiency of its competitors.

In this study, we combined local species abundances and habitat modeling, using local and landscape features, to understand how forest management intensity influences the response of co-occurring species to the environment. Specifically, we aimed at answering two questions: (1) how does species co-occurrence influence species’ habitat selection and (2) how does forest management intensity modify species’ habitat selection in different landscape contexts?

Many forest species are negatively impacted by intensive forest management [50–53] but can still persist in situations where interspecific interactions allows them to diversify their habitat use [54–56]. We relied on multi-species modeling to incorporate interspecific interactions [11] and restricted our analysis to cavity-nesting and canopy-foraging bird species, which allowed us to assume direct interspecific interactions concerning nesting and foraging sites during the breeding season. In this way, we could account for possible associations among species (as for research question 1), and for those species directly relying on structures which are influenced by forest management (as for research question 2). We modeled the abundance responses to the environment of co-occurring and potentially associated birds of the cavity-nesting and canopy-foraging guilds, accounting for habitat structure, management intensity, availability of tree-related microhabitats (TreMs) [57], and landscape composition. We expected that the response of each species to the habitat structure in presence of associated species would deviate from the responses excluding association among species. Although this would not allow us to determine causal links, it may indicate the presence of an effect, signaled by the direction and magnitude of the correlations between species’ abundances. Regarding the landscape context, we expect that in suboptimal landscapes, species will broaden their habitat choice where management intensity is higher, as a result of higher occurrence rates in suboptimal sites.

## Results

### Guilds abundance and associations

**Fig. 1.**
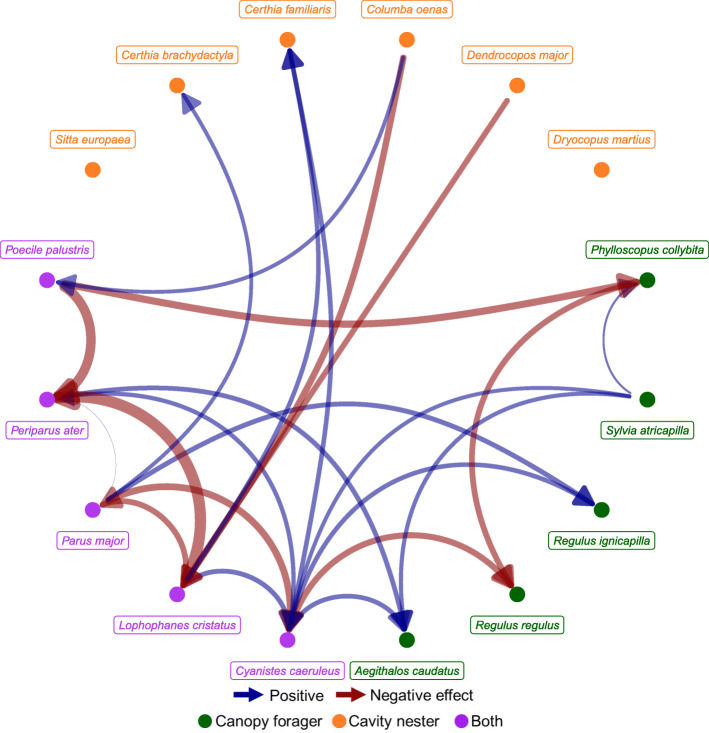
Statistical associations among species found by modeling species abundances as a function of co-occurring species and environmental predictors. Twenty-two positive and negative associations were found. Associations are considered as density-dependent effects on the abundance of the influenced species, possibly due to interspecific competition over, in this case, nesting and feeding resources. Arrow width indicates the effect size

Over 3 years, we recorded 8812 individuals belonging to 16 different species that were detected at least 30 times, of which 11 were cavity nesters, 10 canopy foragers, with 5 belonging to both guilds (Table 1). The most common species counted at plots was the coal tit (*Periparus ater*), with an average number of individuals per plot and visit of 0.93 (± 0.84 SD), while the short-toed treecreeper (*Certhia brachydactyla*) was the least common (0.02 ± 0.17 SD). The multi-species model included 77 assumed associations between the 16 species (figure S1). Species that were not influenced by other species included woodpeckers, large-sized species (e.g., stock dove *Columba oenas*), species that can substantially rely on other resources (e.g., blackcap *Sylvia atricapilla* forage also in shrubs), or species that can escape competition by various means (e.g., European nuthatch *Sitta europaea* can substantially modify the cavity opening). The mean probability of detection across all species was 0.18, ranging from 0.03 (± 0.69 SD) of the blackcap to 0.51 (± 0.55 SD) of the great tit (*Parus major*). All 16 species responded in abundance to at least one forest variable, reporting a credible estimate (*f* > 0.9), while for ten species the effect of the forest management intensity index scored a credible and negative estimate (Table 1). Co-occurring species scored credible effect estimates with up to four other species, totaling between the two guilds 22 credible associations, out of the 77 hypothesized (Fig. 1). Statistical associations indicated thirteen negative and nine positive effects between two species. Three species, the great tit, the marsh tit (*Poecile palustris*), and the short-toed treecreeper, returned only one association, while four associations were found for the blue tit (*Cyanistes caeruleus)*.

**Table 1:** Effect estimates (with standard deviations) of environmental predictors and associated species on species abundances considered in the study belonging to either the cavity nesting or canopy foraging guild

Species	Forest variables						Associated			
	BA	TreM richness	TreM abundance	ForMI	Conifer share	Altitude	Species 1	Species 2	Species 3	Species 4
*Columba oenas*	− 0.28 (0.21)			− 0.17 (0.154)		− 0.63 (0.6)	NA	NA	NA	NA
*Dendrocopos major*	− 0.33 (0.17)					− 0.71 (0.35)	NA	NA	NA	NA
*Dryocopus martius*	− 0.28 (0.2)			− 0.17 (0.159)		− 1.07 (0.61)	NA	NA	NA	NA
*Phylloscopus collybita*	− 0.36 (0.16)		− 0.16 (0.13)	− 0.13 (0.092)			*P. palustris* − 0.08 (0.05)	*S. atricapilla* 0.16 (0.04)		
*Sylvia atricapilla*	− 0.39 (0.15)						NA	NA	NA	NA
*Regulus ignicapilla*	− 0.25 (0.16)				− 0.71 (0.3)		*C. caeruleus* − 0.08 (0.05)	*P. major* 0.14 (0.11)		
*Regulus regulus*				− 0.17 (0.088)	0.73 (0.3)	− 0.4 (0.26)	*C. caeruleus* − 0.06 (0.04)	*P. collybita* − 0.03 (0.02)		
*Aegithalos caudatos*	− 0.32 (0.2)	0.79 (0.44)				− 1.92 (0.74)	*C. caeruleus* − 0.23 (0.15)	*P. ater* − 0.24 (0.12)	*S. atricapilla* 0.21 (0.11)	
*Cyanistes caeruleus*					− 2.32 (0.75)	− 1.93 (0.74)	*L. cristatus* 0.37 (0.21)	*P. major* 1.3 (0.33)	*P. ater* − 0.25 (0.13)	*S. atricapilla* − 0.19 (0.12)
*Lophophanes cristatus*	− 0.27 (0.16)			− 0.17 (0.092)			*C. oenas* − 0.61 (0.26)	*D. major* − 0.1 (0.05)		
*Parus major*	− 0.25 (0.16)			− 0.13 (0.089)		− 0.58 (0.29)	*L. cristatus* − 0.15 (0.06)			
*Periparus ater*						1.27 (0.46)	*L. cristatus* 0.29 (0.09)	*P. major* *− 4 (0.72)*	*P. palustris* − 0.11 (0.09)	
*Poecile palustris*	− 0.28 (0.2)			− 0.17 (0.124)	− 1.7 (0.73)	− 0.74 (0.58)	*C. oenas* 0.44 (0.21)			
*Sitta europaea*	− 0.26 (0.17)			− 0.22 (0.12)	− 0.73 (0.33)		NA	NA	NA	NA
*Certhia brachydactyla*	− 0.29 (0.21)		− 1.13 (0.69)	− 0.21 (0.161)		− 3.05 (0.96)	*P. major* 0.92 (0.33)			
*Certhia familiaris*	− 0.23 (0.17)			− 0.14 (0.087)			*L. cristatus* 0.12 (0.05)	*C. caeruleus* − 0.043 (0.035)		

*BA* basal area, *ForMI* forest management intensity index, *NA* not applicable to species considered not to be influenced by other species due to life history traits (see “Methods” and Additional file 1)

### Habitat characterization

**Fig. 2.**
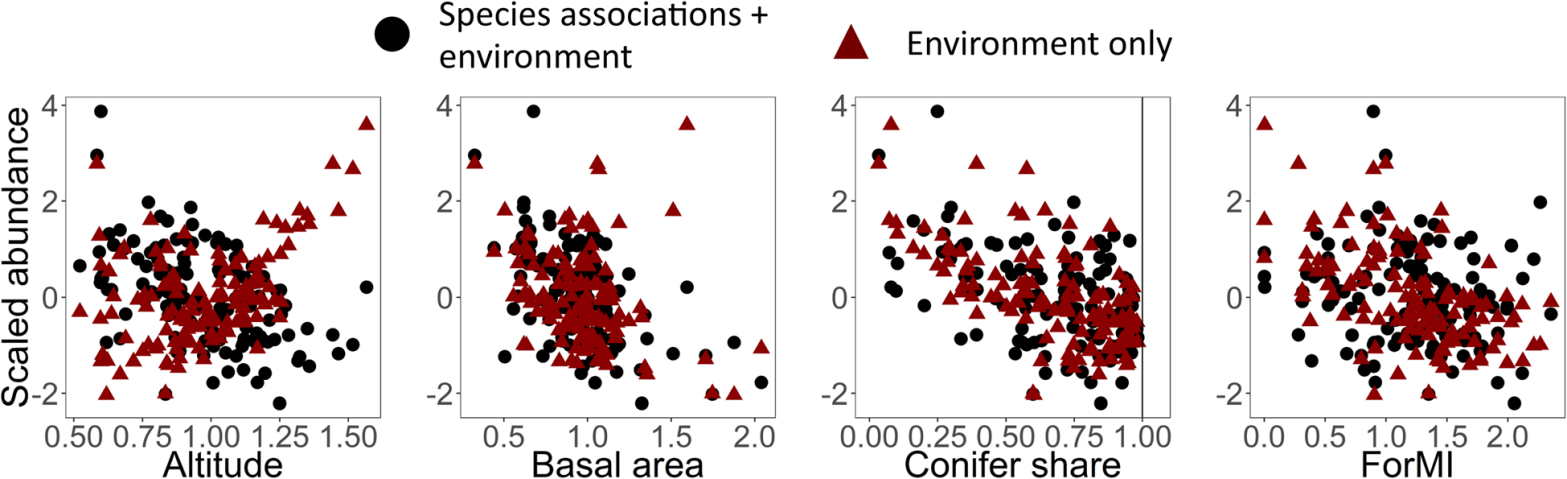
Scaled abundance of the bird assemblage including cavity nester and canopy forager guilds estimated from 127 forest plots in the Black Forest, Germany. Red triangles show forest plot posterior abundance means as a function of environmental predictors, while black circles show means incorporating also the statistical associations among species

**Fig. 3.**
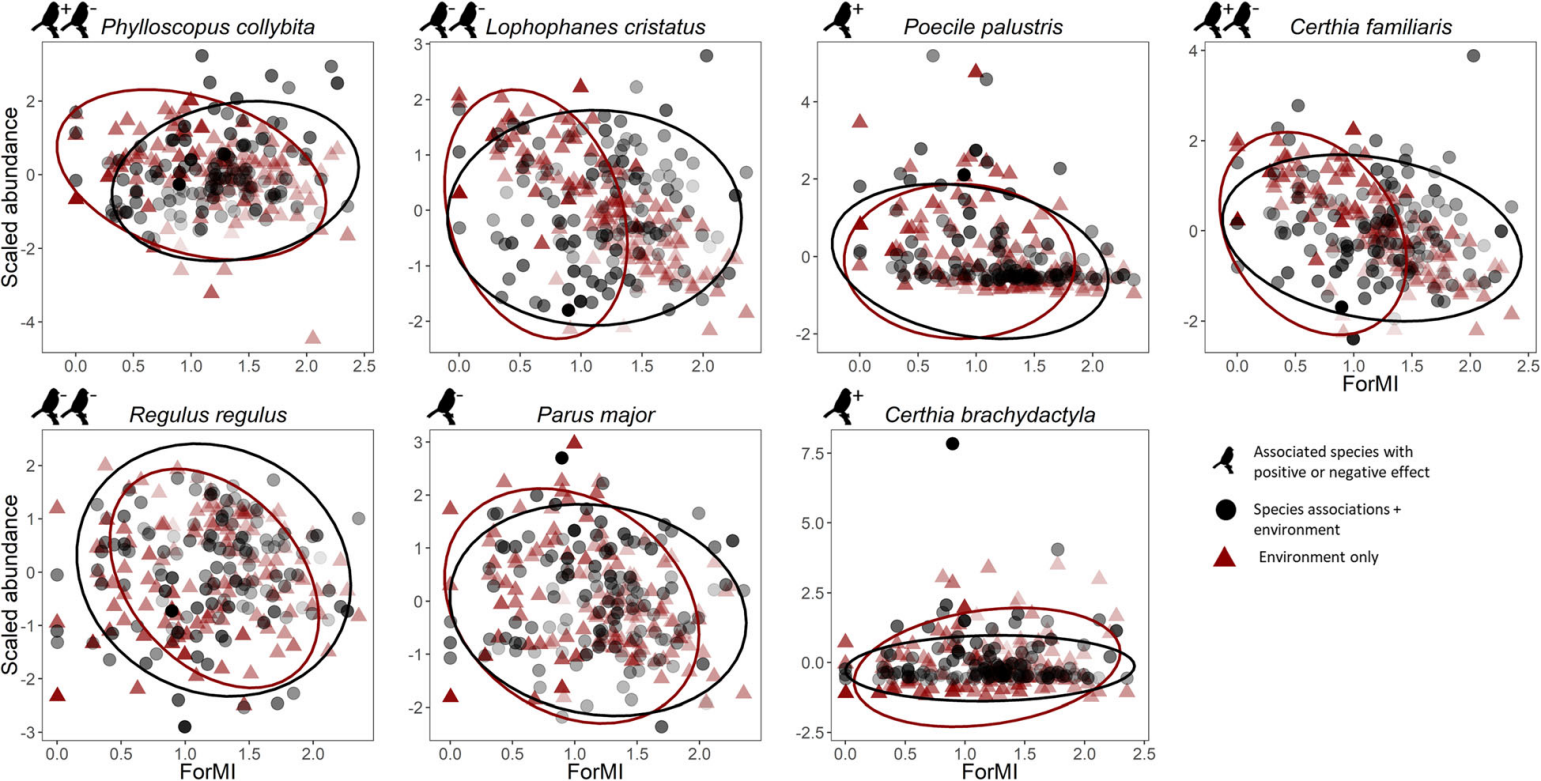
Scaled abundance of the species influenced by forest management intensity index (ForMI). Red triangles show forest plot posterior abundance means as a function of environmental predictors, while black circles show means incorporating also the statistical associations among species. 95% ellipses coded with same colors encircle the section along the environmental gradient where the upper quartile of the abundances occurs. Bird icons indicate the number of associated species with positive (+) and negative (-) effects

For the entire studied assemblage, we did not find differences in abundance along the gradients of forest variables, when including species associations in the abundance model (Fig. 2). Along the altitudinal gradient, the abundances were also similar in both models, although the model without associations presented a rather positive response to altitude, due to the high abundances estimated for the coal tit. However, absolute abundances per species and plot covered a larger environmental gradient in the model accounting for associations: abundances were spread along a larger ForMI gradient (Fig. 3). This was particularly evident for the crested tit (*Lophophanes cristatus*) and the Eurasian treecreeper (*Certhia familiaris*), despite both having negative associations with other species.

Based on the forest variables identified as abundance predictors, the hierarchical clustering identified 79 plots as belonging to high management intensity class (HMI) plots, with means of basal area = 35.66 (± 7.87 SD), conifer share = 0.76 (± 0.20 SD), ForMI = 1.47 (± 0.36 SD), TreM richness = 8.04 (± 2.72 SD), and TreM abundance = 30.38 (± 14.39 SD). Low management intensity (LMI) plots were 48 with means of basal area = 37.88 (± 12.64 SD), conifer share = 0.51 (± 0.25 SD), ForMI = 0.78 (± 0.44 SD), TreM richness = 14.5 (± 4.49 SD), and TreM abundance = 67.4 (± 48.41 SD). Altitudinal distribution was similar between management intensity classes. Landscape variables were not correlated with management intensity classes, with all perMANOVAs scoring *p* > 0.05.

**Fig. 4.**
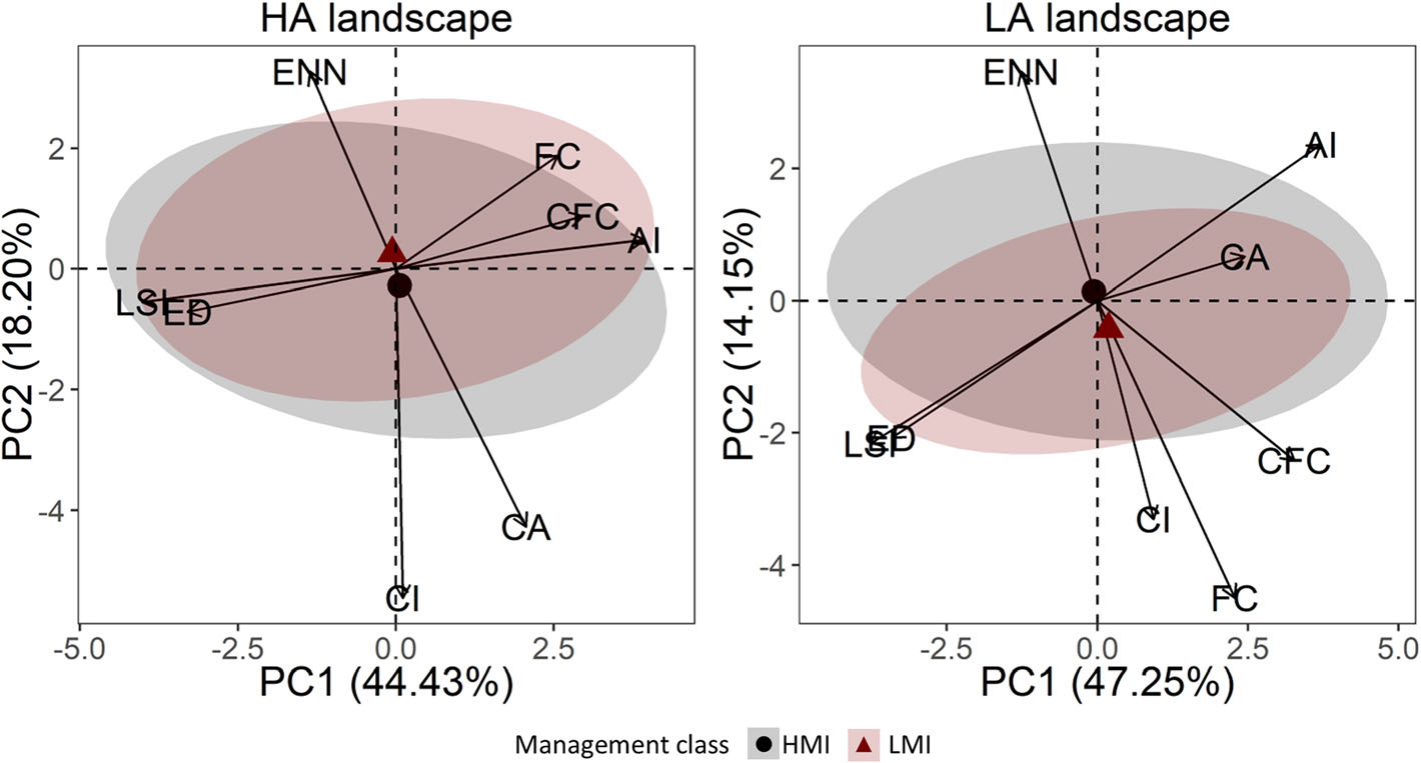
Multivariate space of the bird assemblage of the Black Forest, Germany, including cavity nesters and canopy foragers, for high (HA) and low (LA) abundance landscapes. Arrows identify the relative landscape variables used to build the PCA axis. Numbers on axis represent the variance explained by the principal components. High management intensity (HMI) and low management intensity (LMI) plots are determined by a hierarchical clustering on forest variables and visualized with 95% confidence ellipses, including the centroids. AI, aggregation index; CA, core area; CI, contiguity index; CFC, conifer forest cover; ED, edge density; ENN, Euclidean nearest neighbor; FC, forest cover; LSI, landscape shape index

**Fig. 5.**
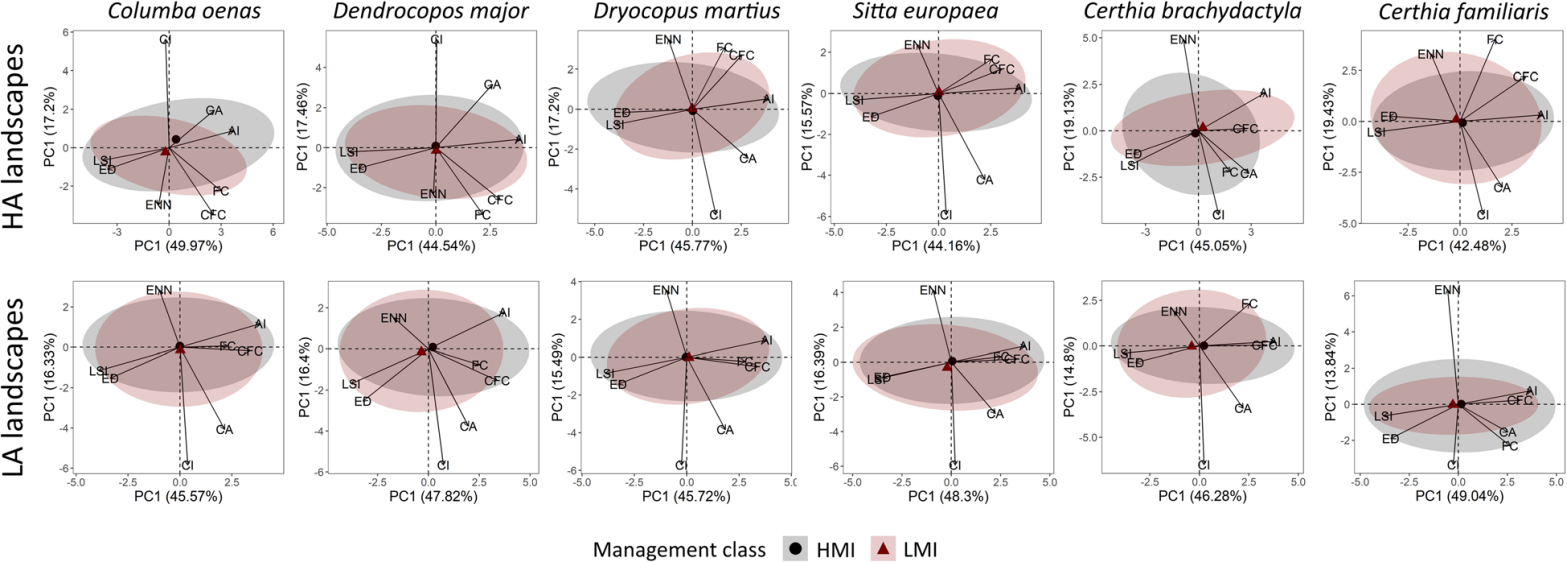
Multivariate space of cavity nesting species (excluding canopy foragers), for high (HA) and low (LA) abundance landscapes, respectively. Arrows identify the relative landscape variables used to build the PCA axis. Numbers on axis represent the variance explained by the principal components. High management intensity (HMI) and low management intensity (LMI) plots are determined by a hierarchical clustering on forest variables and visualized with 95% confidence ellipses, including the centroids. AI, aggregation index; CA, core area; CI, contiguity index; CFC, conifer forest cover; ED, edge density; ENN, Euclidean nearest neighbor; FC, forest cover; LSI, landscape shape index. From left to right, species are stock dove, great spotted woodpecker, black woodpecker, European nuthatch, short-toed treecreeper, Eurasian treecreeper

**Fig. 6.**
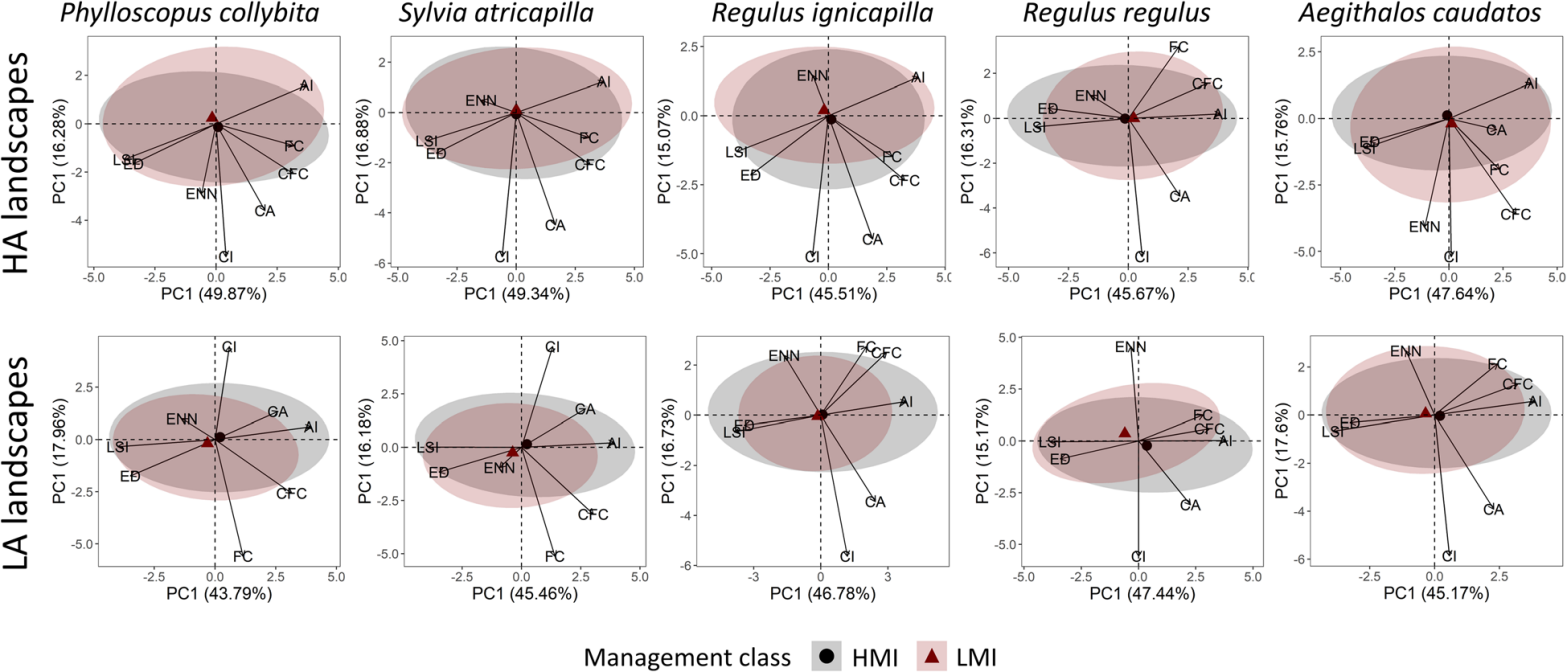
Multivariate space of canopy foraging species (excluding cavity nesters), for high (HA) and low (LA) abundance landscapes, respectively. Arrows identify the relative landscape variables used to build the PCA axis. Numbers on axis represent the variance explained by the principal components. High management intensity (HMI) and low management intensity (LMI) plots are determined by a hierarchical clustering on forest variables and visualized with 95% confidence ellipses, including the centroids. AI, aggregation index; CA, core area; CI, contiguity index; CFC, conifer forest cover; ED, edge density; ENN, Euclidean nearest neighbor; FC, forest cover; LSI, landscape shape index. From left to right, species are chiffchaff, European blackcap, firecrest, goldcrest, long-tailed tit

**Fig. 7.**
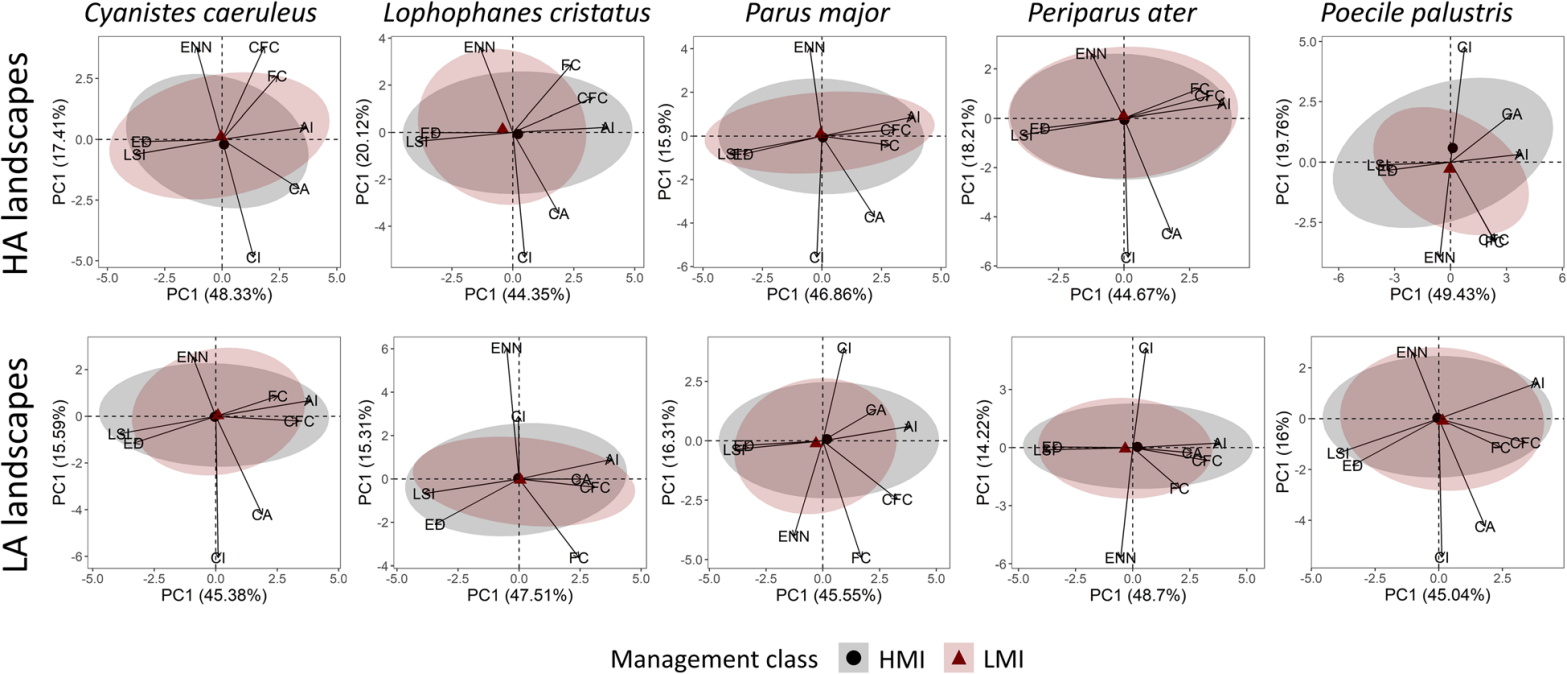
Multivariate space of species belonging to both cavity nesting and canopy foraging guilds, for high (HA) and low (LA) abundance landscapes. Arrows identify the relative landscape variables used to build the PCA axis. Numbers on axis represent the variance explained by the principal components. High management intensity (HMI) and low management intensity (LMI) plots are determined by a hierarchical clustering on forest variables and visualized with 95% confidence ellipses, including the centroids. AI = aggregation index; CA, core area; CI, contiguity index; CFC, conifer forest cover; ED, edge density; ENN, Euclidean nearest neighbor; FC, forest cover; LSI, landscape shape index. From left to right, species are blue tit, crested tit, great tit, coal tit, marsh tit

The PCA of the landscape variables for high abundance (HA) landscapes of the entire bird assemblage could explain ~ 63 % of the observed variation. The first component scored positive loadings for aggregation index, forest and conifer forest cover, and negative loadings for edge density and landscape shape index. This suggests that high abundances are observed in landscapes with large, contiguous forest patches (Table 2). Low abundance (LA) landscapes differed in the loadings of contiguity index, core area, forest, and conifer forest cover. This indicates that low abundances are to be found in landscapes with less forest cover and smaller forest patches. In this case the variance explained was ~ 61 % (Table 2). Confidence ellipses showed that the bird assemblage of HA landscapes occurred in HMI and LMI plots with a similar landscape structure. In contrast, LMI plots from LA landscapes were characterized by bird assemblages occurring in narrower landscape condition than HMI plots. This indicates that the assemblage occurred across a wider set of landscape conditions in suboptimal landscapes under high management intensity (Fig. 4). By looking at the position and width of the confidence ellipses for the single species, a similar pattern was observed within the cavity-nesting and canopy-foraging guilds, especially among those showing species associations (Figs. 5, 6, and 7). Specifically, Eurasian treecreeper (*Certhia familiaris*), chiffchaff (*Phylloscopus collybita*), European blackcap (*Sylvia atricapilla*), firecrest (*Regulus ignicapilla*), goldcrest (*Regulus regulus*), blue tit (*Cyanistes caeruleus*), and coal tit (*Periparus* ater) occurred over a broader range of landscape conditions in HMI plots located in LA landscapes. Among the other species, stock dove (*Columba oenas*) and marsh tit (*Poecile palustris*) showed an opposite pattern, with broader ranges of landscape conditions in HMI plots from HA landscapes. The woodpeckers (*Dendrocopos major* and *Dryocopus martius*), short-toed treecreeper (*Certhia brachydactyla*), great tit (*Parus major*), and crested tit (*Lophophanes cristatus*) used broader ranges of landscape conditions in HMI plots for both landscape categories. Finally, the occurrence patterns of European nuthatch (*Sitta europaea*) and long-tailed tit (*Aegithalos caudatus*) were similar in all landscapes.

**Table 2:** Factor loadings of the first two components (PC) of the principal component analysis on the landscape variables for high abundance (HA) and low abundance (LA) landscapes. Variance explained by each component is in brackets

Landscape variable	HA landscape		LA landscape	
	PC1 (44.43 %)	PC2 (18.20 %)	PC1 (46.29 %)	PC2 (14.93 %)
Aggregation index	0.49	0.06	0.46	0.30
Contiguity index	0.01	− 0.68	0.12	− 0.41
Core area	0.26	− 0.53	0.31	0.08
Edge density	− 0.41	0.09	− 0.43	− 0.25
Euclidean nearest neighbor distance	− 0.17	0.41	− 0.16	0.43
Landscape shape index	− 0.50	− 0.07	− 0.47	− 0.27
Forest cover	0.32	0.23	0.29	− 0.56
Conifer forest cover	0.37	0.11	0.41	− 0.30

## Discussion

### Habitat use of co-occurring species

The role of interspecific associations in modulating how species use their habitat depends on the context in which they occur. Environmental, spatial, temporal, or community settings may all alter the magnitude and direction of such associations [58]. Large-scale environmental drivers have previously been linked with alterations in species association and co-occurrence patterns [59]. In our study, using forest birds, we demonstrated that the occurrence and abundance of species at a small (plot level) spatial scale are linked to local environmental drivers and, simultaneously, to co-occurring species. Hence, we observed a context-dependent change in habitat selection of species. Although our approach did not identify interactions empirically confirming the causation, it showed that, apparently, species associations can influence the local abundance of species, in addition to their environment-related baseline occurrence [11]. Our approach could evaluate only unidirectional effects between species pairs. Hence, we could not test all possible association effects, which may be reciprocal in some instances [40]. Another limitation of our model is that it did not evaluate potential interaction effects among species included as covariates. This should be addressed in future studies. We, also, did not include potential predators affecting the targeted species, another density-dependent process that may strongly affect habitat selection [9, 60]. However, many species within the guilds studied are similar in size and life history and have mutualistic anti-predatory relationships [61].

As an example of how birds' habitat preferences were modulated by co-occurrences, we discuss the Eurasian treecreeper. According to our results concerning only forest variables, we would predict lower abundances at sites with high management intensity. However, the positive association between the crested tit and the Eurasian treecreeper predicts the latter to still persist in higher abundances at intensively managed sites. The mechanism behind the observation that increasing numbers of crested tits are associated with an increasing abundance of the Eurasian treecreeper is likely rooted in the network of relationships within the entire bird assemblage. One possible explanation could be that crested tit and treecreeper neither compete for food nor nest sites but may help each other to avoid predation and thereby elevate survival by participation in mixed-species flock in winter. However, we did not directly observe that, but rather interpreted statistical associations as direct or indirect influence of a species on another one, causing the abundance of the species to change according to density-dependent processes, e.g., competition over resources [62]. Moreover, competitor species density can also cue other species about habitat quality and indirectly drive their local habitat selection [63].

Most of the species association effects were less important than forest variable effects, indicating that the primary determinant of species abundance remains the habitat structure. However, habitat structure cannot fully explain the processes of habitat selection and community assembly, without considering it alongside with other processes, such as competition or predation [1, 60]. Compelling evidence shows that it is not possible to separate the effect of environmental filtering from that of biotic interactions in traditional study settings [2]. Indeed, we observed that the relative abundances of co-occurring species are related to each other and, together with the environment, modulate the response of species to environmental gradients (environmental filtering). This raises the question whether interspecific associations play a similarly important role within forest bird guilds other than those investigated, whenever the set of resources they use is limited by forest management.

The effect of species associations sheds new light on the effect of forest structure on the targeted bird species. Conifer share at the plot scale influenced abundances of species mainly in a negative way, agreeing with other studies from the same [64] and other regions [65]. The effects of basal area and ForMI were mainly negative among species, despite different degrees of habitat specialization among species and the fact that management intensity is only moderately related to forest structure in our plots [66]. Still, specialized species responded negatively to management intensity, which is common among cavity nesters [43, 44]. Instead, the occurrence rates of forest generalist species, such as the great tit, are often associated with increasing management intensity [67]. Nonetheless, we observed a negative response to management intensity also for this species. The great tit is often found in coexistence with other members of the family Paridae, which may indicate mutualistic relationships [68, 69]. This coexistence is based on different types of resource partitioning leading to unique combinations of niche characteristics [47, 62]. In particular, positive interactions among co-occurring species can be relevant in suboptimal habitats, where some species may facilitate the habitat use of others [70]. This becomes particularly evident outside the breeding season, when mixed-species flocking within the same foraging guild can improve the protection against predators and feeding efficiency [71]. Hence, we stress that positive responses to high management intensity may depend also on the identity of co-occurring species.

### Management intensity modifies the habitat preferences according to the landscape context

Birds can exhibit larger territory areas, lower occupancy rates, and lower abundances in suboptimal habitats [72–75]. In landscapes characterized by low abundances of forest birds (LA landscapes in our study), species may simply be forced to exploit a broader range of environmental conditions. Our analysis showed that some species (e.g., Eurasian treecreeper) occurred in high management intensity (HMI) plots across a wider set of environmental conditions (at the landscape scale), especially in suboptimal landscapes (i.e., with lower abundances). Previous studies related higher occurrence rates with more generalist habits in birds [25, 76]. Hence, populations of the same species could also increase their occurrence rate, by broadening their habitat preferences. Another study focusing on boreal forest birds provided findings similar to ours, indicating that the local habitat structure is a more important driver of niche breadth rather than landscape structure [19]. Species with and without interspecific associations showed similar abundance patterns across all landscapes, and the responses to environmental predictors among species were consistent with each other. Therefore, we can provide support to the statement that management intensity can modify the habitat structural preferences of forest birds, with the remark that this is more evident in suboptimal landscapes. Low abundances may be a result of habitat fragmentation, suboptimal habitat structure, disturbance, or other factors [77]. In this study, low abundances were found in landscapes characterized by smaller forest patch size and less forest cover. In fact, the landscape context may act as precondition for a species to utilize different environments [78, 79]. Moreover, studies focusing only on foraging guilds and including old-growth forests (not represented in our study) associated wider niche breadths with more pristine forests [80, 81]. This is partly in accordance with our results in HA landscapes and LMI plots for Eurasian treecreeper, chiffchaff, and long-tailed tit. This showcases a great difficulty in optimizing conservation interventions for the widest possible array of species in forest management strategies targeting the conservation of forest bird species [82]. This is especially true for cavity nesters, the guild dominated by resident species with many of them being in jeopardy in managed forests in Europe [83–85]. It has been demonstrated that forest management may, through reducing niche diversity, largely affect the ability of closely related species and competing species to coexist [60].

## Conclusions

Interspecific associations can result in striking differences in habitat selection for bird species with overlapping habitat preferences [49, 86]. This highlights the plasticity of birds in their habitat use, and in their ability to exploit different habitat conditions. We showed that within the forest bird assemblage, species can display different habitat preferences according to the presence and abundance of co-occurring and associated species. This study suggests that interspecific associations result in higher abundances of some bird species in more intensively managed forests, which may be a consequence of factors beyond the local habitat structure. Hence, to design management actions based exclusively on species-habitat relationships may not necessarily deliver the best possible results, and managers should be aware of potential effects of interspecific associations. At the same time, in suboptimal landscapes species are forced to exploit a broader range of environmental conditions in intensively managed forests, which may hamper the effectiveness of conservation-oriented management wherever landscape-scale factors are not considered.

## Methods

### Study area

The study was performed in 127 1-ha plots located in the forest landscape of the Black Forest, southwestern Germany, (latitude 47.6°–48.3° N, longitude 7.7°–8.6° E; WGS 84). Plots were selected in the framework of the ConFoBi Research Training Group ([87]; confobi.uni-freiburg.de). Selection focused on stands with tree age over 60 years and located at least 1 km from each other. A stratified-random selection was then applied along two environmental gradients, representing the local habitat structure (number of standing dead trees per plot) and the landscape context (forest cover in the 25 km^2^ surrounding the plot), respectively. The forest management practiced in the study area consists of single tree selection under close-to-nature forest management leading to continuous cover forests [88, 89]. The plots ranged, in terms of elevation, between 443 and 1334 m a.s.l., and represented a typical temperate mixed mountain forest, dominated by Norway spruce (*Picea abies*) (42.8%), silver fir (*Abies alba*) (18.5%), and European beech (*Fagus sylvatica*) (15.3%). Due to the forest management history of the study area, conifer and mixed-conifer forests occur throughout the above elevation range, also outside of their natural altitudinal distribution.

### Environmental predictors

The environmental descriptors included in the analysis were collected at the plot scale (1 ha), containing forest structure data, and at the landscape scale (up to 5 km^2^), including several landscape configuration metrics and forest cover. The spatial scale of each predictor reflected both the overall study design [87] and the extents at which bird-related ecological processes are usually investigated [90–93].

#### Forest variables

The forest structure of the plots was described in terms of tree basal area, share of conifers, richness and abundance of TreMs [94], an index of forest management intensity [95], tree species composition and deadwood volume. An inventory used to describe forest structure comprised species identity and diameter at breast height (DBH) of all living trees (with DBH > 7 cm), from which basal area and the share of conifers were derived. In addition, the DBH of all snags (with DBH > 7 cm and height > 1.3 m) on the plots was measured. Lying deadwood data was collected using the line intersect method, consisting in walking a V-transect from the north-east corner to the center of the southern plot border to the north-west corner of each plot and counting all deadwood intersecting the transect [96]. The abundance and richness of TreMs was retrieved from previous research in the same plots [94]. TreMs are considered to be “a distinct, well delineated structure occurring on living or standing dead trees, that constitutes a particular and essential substrate or life site for species or species communities during at least a part of their life cycle to develop, feed on, using as shelter or to breed” [57] and have shown correlations to the richness and abundance of forest-dwelling vertebrates and (saproxylic) insects [5, 6, 97]. TreMs are usually grouped into seven forms, including cavities, tree injuries and exposed wood, crown deadwood, excrescences, fruiting bodies of saproxylic fungi and slime molds, epiphytic, epixylic and parasitic structures, and fresh exudates such as sap run and heavy resinosis [98]. The forest management intensity index (ForMI), calculated from forest variables, measured three different management aspects [95]: (a) the proportion of harvested tree volume compared to the maximum volume, (b) the proportion of tree species not belonging to the natural species composition, and (c) the ratio of artificial (showing signs of cutting) vs. natural deadwood. The index spans values 0–3, where 0 would indicate a forest not managed for timber production and 3 an intensively managed production forest. In addition, the average altitude of each plot was provided from a digital terrain model with spatial resolution of 0.5 m (State Office for Geoinformation and Land Development Baden-Württemberg, Germany).

#### Landscape variables

The landscape-scale predictors included the forest cover-based metrics describing the fragmentation of the forest surrounding the plots. Forest cover was derived from the land cover map provided by the State Office for Geoinformation and Land Development of Baden-Württemberg, Germany (Geobasdata ©, www.lgl-bw.de, ref. no: 2851.9-1/19). Forest cover was assessed in the neighboring five km^2^ circular area, separately for conifer and total forest. The landscape metrics were computed on the same land cover map, using only areas classified as forest for the computation (i.e., binary map), and employed the software FRAGSTATS [99]. We considered six metrics commonly employed to describe fragmentation and patchiness of the landscape: the aggregation index, the contiguity area index, the core area index, the edge density, the Euclidean nearest neighbor, and the landscape shape index. These metrics were selected because either evidence or experts suggest they have an effect on the numerical response of birds [90–93].

### Bird sampling

Birds were sampled using standardized point counts with limited distance of 50 m, each conducted from the plot center. Point counts were repeated up to three times/year during the period March–June in years 2017–2019 (i.e., encompassing most of the breeding season), starting half an hour after sunrise with the latest sample collected at 12:00 CET. Each survey at each plot lasted 20 min, during which bird individuals were recorded repeatedly every 5 min, in order to reach a reasonable sample coverage [100, 101].

Each bird species was classified as cavity nester, insectivorous canopy forager, or both according to standard references [28, 102, 103]. We adopted the rule of thumb for skewed distribution and excluded species recorded less than 30 times in the entire study from the analysis, to trade-off between number of species and robustness of the analysis. To estimate the effect of the co-occurrence of a given species on a potentially associated one, we established linkages between species, i.e., assumed that the abundance of a species is correlated to the abundance of another one. To inform the direction of the linkage, i.e., whether species A is presumably affecting species B or the opposite, we relied on the existing literature (see Additional file 1, Table S1) [28–30, 34, 35, 37–46, 48, 49, 69, 102, 104–113].

### Abundance estimates

To account for co-occurrence effects on species responses to habitat structures, bird abundance was estimated using community N-mixture models [114, 115]. Such models allow for estimating the abundance of species belonging to an assemblage as a function of environmental predictors, considering the detectability error by employing count data from repeated surveys. Our models incorporated the density-dependent effect of co-occurring and potentially associated species (Additional file 2). Species were paired according to their relationship (as described in Additional file S1), and assumptions about the direction of the relationship were not made. That is, after establishing from the literature that the species A is presumably affecting species B, and hence their abundances are correlated, we did not assume this correlation to be positive or negative, but only density-dependent. We restricted our analysis by focusing only on the cavity nester and canopy forager species found in our study area, which potentially compete over resources. Hence, if a relationship was present, the abundance of species A was considered a covariate of the response variable, i.e., the abundance of the species B, similarly to other research on species co-occurrences [116]. Species abundance was modeled as a Poisson process, while the detectability was modeled as a binomial distribution, dependent on the abundance process and moderated by the date and time of each survey, to model individual heterogeneity in detectability. The forest variables included in the species abundance model had a variance inflation factor ≤ 3, indicating that multicollinearity was not an issue. All predictors were scaled prior to analysis. The full model was built in JAGS programming language and fitted by applying Bayesian inference. We used uninformative priors and ran three MCMC chains of 400,000 iterations, discarding the first 10,000 and thinning by 90. We considered reliable model parameter estimates those drawn from a posterior distribution where the proportion with the same sign as the mean was *f* ≥ 0.9. If this was not the case, the model parameter was discarded from the analysis and the model ran again. We considered that chains reached convergence when the Gelman-Rubin statistic (r-hat) was ≤ 1.1 for all parameters [117]. All analyses were conducted in the R statistical environment. The community model was built with the package “jagsUI” [118]. Model posterior means including associations were compared against the means of the model without association using 95% confidence ellipses.

### Habitat characterization

To account for the effect of management intensity on species’ responses to habitat at local scale, the forest structure of each plot was characterized by performing hierarchical clustering of the forest variables, aimed at grouping them in two clusters of high management intensity (HMI) and low management intensity (LMI) plots. Each plot was assigned to a category of management intensity using the K-means clustering method on the forest variables. At the landscape scale, instead, we characterized the landscape structure by building two gradients using the first two components of a principal component analysis (PCA) on the correlation matrix of landscape variables. Considering that species perceive the landscape according to their life histories, we performed PCAs on the guilds and on each species. More or less fragmented landscape configurations can result in very different bird abundances [72, 119]. Therefore, we considered optimal landscapes as those where the estimated abundance at plot level was higher than the mean abundance observed across the study area (0 after scaling). In this way, we characterized both the landscape with high (HA) and low (LA) abundances, as proxies for optimal and suboptimal-to-unsuitable landscapes, and each included both HMI and LMI plots. We performed a permutational Multivariate ANalysis Of VAriance (perMANOVA), to test whether each landscape variable associated to each plot differed among management intensity classes to further confirm that landscape structure was independent from forest structure. Then, we compared the habitat structure for HMI and LMI plots along the landscape gradients in LA and HA landscapes. We calculated the abundance of each guild by summing up the respective species’ estimated abundances and scaling them. The habitat structure was visualized by plotting the 95% confidence ellipses of abundance estimates and visually comparing the respective position and width. The R package ‘vegan’ was employed for the analysis [120].

## Supplementary Information

**Additional file 1 **Species classification in the cavity nester and canopy forager guilds. Based on the reference, we established 77 potential associations. **Figure S1.** Potential relationship between species identified or suggested in the literature. **Table S1.** Species associations derived from the literature, experts’ comments or inferred from body size and closely-related species

**Additional file 2.** Multi-species abundance model incorporating the species association process.

## Data Availability

The dataset supporting the conclusions of this article is available in the ConFoBi database repository, http://confobi-db.vm.uni-freiburg.de/geonetwork/srv/ger/catalog.search#/home
