# Supplementary material for: Species co-occurrence and management intensity modulate habitat preferences of forest birds
Source: BMC Biol. 2021 Sep 23;19:210. doi: 10.1186/s12915-021-01136-8 (PMC8459526; doi:10.1186/s12915-021-01136-8)
Supplement: Supplementary file 1 — Additional file 1 Species classification in the cavity nester and canopy forager guilds. Based on the reference, we established 77 potential associations. Figure S1. Potential relationship between species identified or suggested in the literature. Table S1. Species associations derived from the literature, experts’ comments or inferred from body size and closely-related species [file 12915_2021_1136_MOESM1_ESM.docx]

**Additional file 1.** Species co-occurrence and management intensity modulate habitat preferences of forest birds

Species classification in the cavity nester and canopy forager guilds. Based on the reference, we established 77 potential associations (figure S1 and table S1).

Cavity nester

*Primary cavity nester*

The primary cavity nesters include species that excavate their own cavity in trees [28], namely the bird family Picidae (Vigors, 1825). The substrate where the cavity is formed triggers competitive interactions among species [29,30]. It is not rare that the same individuals use the same cavity in multiple years, or that the same individuals excavate more than one cavity per year, triggering competition among cavity nesters (including non-bird species) for the occupation of a given cavity. The choice of the nesting cavity is primarily driven by body size, given that some species are simply too large to fit in small cavities, while the quality of the nesting hole, such as the exposure to predation, triggers competitive interactions [40]. Woodpecker cavities can last up to 10 years (median value) in coniferous forests, especially those excavated by large woodpecker species [105], while non-excavated holes have a shorter lifespan (median = 4.4 years) in coniferous forests [106]. The role of cavities in forests has been described using network theory, given the potentially large number of species connected through this key structure [107].

*Secondary cavity nester*

The secondary cavity nesters include species that use cavities generated by natural processes or excavated by primary cavity nesters [28]. The availability of cavities in primeval forests is not a limiting factor for secondary cavity nesters [34]. Instead, this may be the case in managed forests, where species-specific responses might show a high degree of variation depending on the predation risk [40, 41], the relative abundance of large, suitable trees and snags [35, 36], the relative abundance of woodpeckers [37], the forest management type [38], the tree species composition [39], and the presence of invasive species [42]. An abundant family of this guild is Paridae (Vigors, 1825), which includes small songbirds that also mainly forage in the canopy. The breeding ecology of this family, and especially the great and the blue tit (*Parus major* and *Cyanistes caeruleus*), have been the focus of vast research. The larger species are considered dominant in the competition for the nesting cavity, and, even though the smaller species can escape competition by relying on smaller cavities, they can still suffer competition when cavities are limited [40, 108, 109]. The only secondary cavity nester potentially excluded from the competitive dynamics is the Eurasian nuthatch (*Sitta europaea*), given its ability to modify the entrance of the cavity [40].

Canopy forager

The canopy foraging guild include those species which feeding substrate is found in the tree canopy [46]. In this case, competition sparks from the optimal foraging substrate-end (e.g. branches vs. needles in conifer canopy) and the different efficiency of each species to forage [48, 68]. In Europe, it comprises mainly foliage gleaners and seed eaters, and it is also influenced by forest management [43–45]. Other species included in this guild are large-sized species, such as the Eurasian jay (*Garrulus glandarius*). From the composition of this guild, we concluded that only insectivorous foliage gleaners could have interactions with the cavity nesters over the nesting site or the feeding substrate, hence we limited our analysis only to the insectivorous canopy forager guild. In this case, competition is driven by the ability of each species to cope with the local condition and the presence of other species, rather than by a limiting resource. Different part of the canopy (e.g. branches, twigs, or needles) or different forest layers offer multiple alternatives for feeding, with species having partly overlapping niches [110–113]. However, some species, such as the coal tit (*Periparus ater*) are particularly efficient at food exploitation, given some environmental condition, so that they can avoid competition with larger species by depleting the food resource [68, 109, 114]. Other species can also escape competition by feeding on the ground [103].


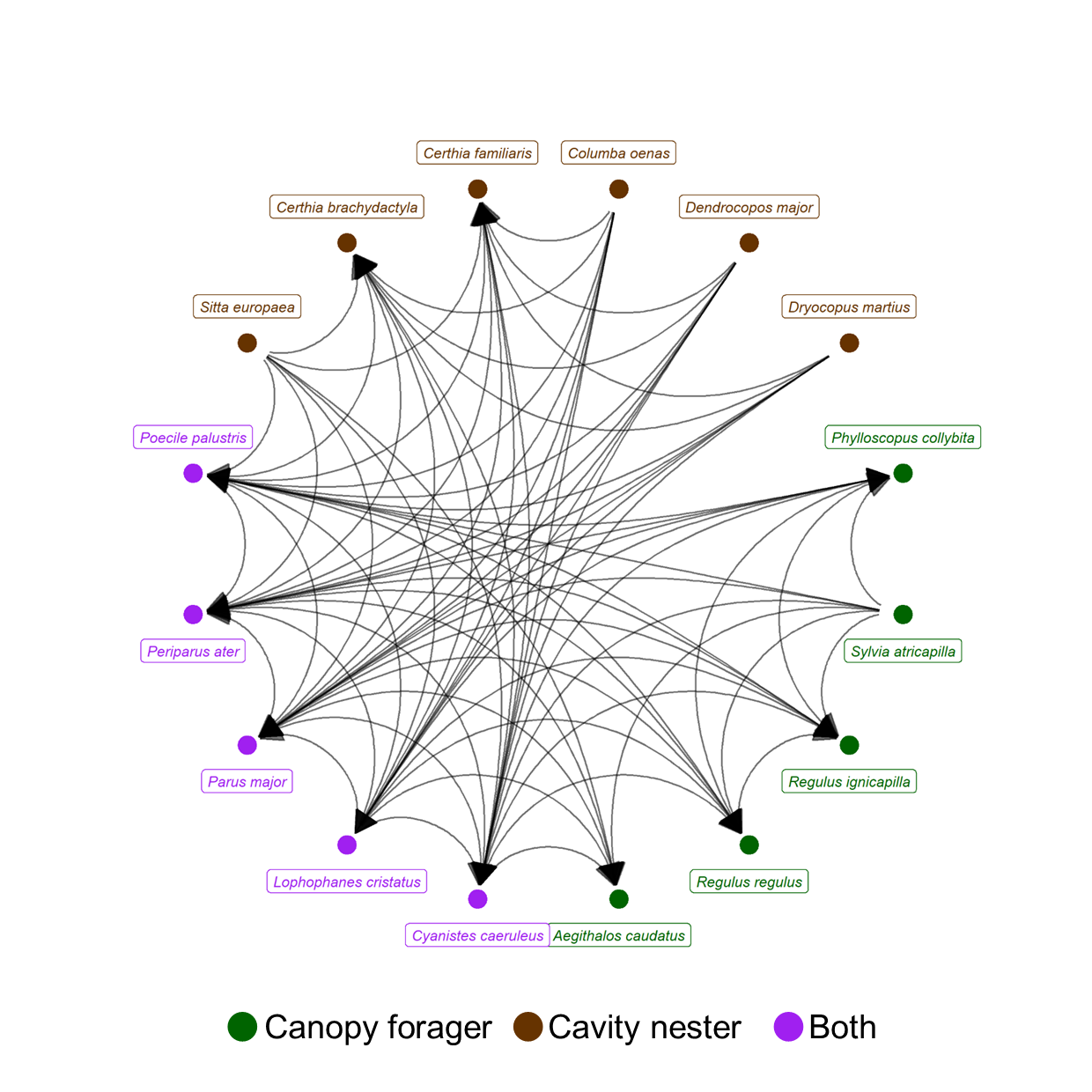


Figure S1: Potential relationship between species identified or suggested in the literature. The arrow points at the species that is influenced. The relationships have been included in the model described in Additional file 2. We interpret the relationships as influence on the abundance, due to competition over resources.

| Table S1: Species associations derived from the literature, experts’ comments or inferred from body size and closely-related species. | | | |
| --- | --- | --- | --- |
| Species | Associated | Reference / reason | |
|  |  | Nesting | Foraging |
| *Columba oenas* | - | Size |  |
| *Dendrocopos major* | - | Primary cavity nester |  |
| *Dryocopus martius* | - | Primary cavity nester |  |
| *Phylloscopus collybita* | *Cyanistes caerulaeus*  *Lophophanes cristatus*  *Parus major*  *Periparus ater*  *Poecile palustris*  *Sitta europaea* |  | [86, 103] |
| *Sylvia atricapilla* | - |  | also ground forager [103] |
| *Reregulus ignicapilla* | *Philloscopus collybita*  *Sylvia atricapilla*  *Regulus regulus*  *Cyanistes caerulaeus*  *Lophophanes cristatus*  *Parus major*  *Periparus ater*  *Poecile palustris* |  | affinity with goldcrest [114] |
| *Regulus regulus* | *Philloscopus collybita*  *Sylvia atricapilla*  *Cyanistes caerulaeus*  *Lophophanes cristatus*  *Parus major*  *Periparus ater*  *Poecile palustris* |  | [86, 114] |
| *Aegithalos caudatos* | *Philloscopus collybita*  *Cyanistes caerulaeus*  *Lophophanes cristatus*  *Parus major*  *Periparus ater*  *Poecile palustris* | size | size |
| *Cyanistes caeruleus* | *Columba oenas*  *Dendrocopos major*  *Dryocopus martius*  *Sylvia atricapilla*  *Lophophanes cristatus*  *Parus major*  *Periparus ater*  *Sitta europaea* | [48, 113] | [86, 110] |
| *Lophophanes cristatus* | *Columba oenas*  *Dendrocopos major*  *Dryocopus martius*  *Sitta europaea* | [111, 112] | [86, 114] |
| *Parus major* | *Columba oenas*  *Dendrocopos major*  *Dryocopus martius*  *Sylvia atricapilla*  *Lophophanes cristatus*  *Poecile palustris*  *Sitta europaea* | [48, 113] | [86, 110, 114] |
| *Periparus ater* | *Columba oenas*  *Dendrocopos major*  *Dryocopus martius*  *Parus major*  *Lophophanes cristatus*  *Poecile palustris*  *Sitta europaea* | [111, 112] | [86, 110, 114] |
| *Poecile palustris* | *Columba oenas*  *Dendrocopos major*  *Dryocopus martius*  *Sylvia atricapilla*  *Lophophanes cristatus*  *Sitta europaea* | size and expert comment | [86] |
| *Sitta europaea* | - | can modify the cavity entrance |  |
| *Certhia brachydactyla* | *Columba oenas*  *Dendrocopos major*  *Dryocopus martius*  *Cyanistes caeruleus*  *Lophophanes cristatus*  *Parus major*  *Periparus ater*  *Poecile palustris*  *Sitta europaea* | size |  |
| *Certhia familiaris* | *Columba oenas*  *Dendrocopos major*  *Dryocopus martius*  *Cyanistes caeruleus*  *Lophophanes cristatus*  *Parus major*  *Periparus ater*  *Poecile palustris*  *Sitta europaea* | size |  |
